# Anticholinergic Burden in Children, Adults and Older Adults in Slovenia: A Nationwide Database Study

**DOI:** 10.1038/s41598-020-65989-9

**Published:** 2020-06-09

**Authors:** Nanca Cebron Lipovec, Janja Jazbar, Mitja Kos

**Affiliations:** University of Ljubljana, Faculty of pharmacy, Ljubljana, 1000 Slovenia

**Keywords:** Health care, Medical research, Risk factors

## Abstract

Anticholinergic burden has been widely studied in specific patient populations with specific conditions. However, the prevalence in the general population is poorly understood. This retrospective cross-sectional study was a nationwide database analysis of outpatient prescriptions of anticholinergic medications. The study was based on Slovenian health claims data of all outpatient prescriptions in 2018. Anticholinergic burden was evaluated using the Anticholinergic Cognitive Burden scale. Three age groups were analysed: children (≤18 years), adults (19–64 years) and older adults (≥65 years). Anticholinergic medications were prescribed to 29.8% of the participants; 7.6% were exposed to a clinically significant anticholinergic burden. The proportion of patients exposed to anticholinergic burden was highest in older adults (43.2%), followed by adults (25.8%) and children (20.7%). The most frequently prescribed medications with the highest anticholinergic activity were antipsychotics and medications for urinary diseases (42.8% and 40.2%, respectively). Medications with second highest activity were mostly antiepileptics (87.3%). Medications with possible anticholinergic activity included diverse therapeutic groups. Anticholinergic burden is highest in older adults but is also considerable among adults and children. Medications with anticholinergic activity belong to diverse therapeutic groups. Further research is needed on safe use of these medications in all age groups.

## Introduction

Many medications have anticholinergic activity, which means they block the neurotransmitter acetylcholine from binding to the muscarinic receptor and hence produce anticholinergic-type effects^1^. Such activity may be desirable, for example in medications for urgent incontinence, or undesirable, as of antipsychotics, antidepressants, and analgesics^2^. The anticholinergic effects range from peripheral, such as dry mouth, constipation, blurred vision, increased heart rate, urinary retention, to central, such as sedation, confusion, dizziness and even cognitive impairment^3^. The concomitant use of multiple medications with anticholinergic activity further increases the likelihood of adverse events^4^.

Anticholinergic burden is referred to the cumulative effect of taking one or more medications with anticholinergic activity^1^. Anticholinergic burden is a strong predictor of cognitive and physical impairment, especially in the elderly population^5^. It has been linked to an increased rate of falls, decline in cognitive function and memory, decline in activities in daily living and higher mortality rates^4,6,7^. Alarmingly, some studies have reported a link between anticholinergic burden and dementia risk^4,8,9^. It has been estimated that approximately 50% of older adults (above 65 years) are exposed to anticholinergic burden^10^. In the context of recently observed rises in the burden of psychiatric illnesses and the increasing trend for polypharmacy, we should expect to see future increases in the use of anticholinergic medications^11^.

Older patients are more likely to experience adverse effects due to age-related changes in pharmacokinetics and pharmacodynamics^3,10,12^. However, children and younger adults can also experience adverse effects of anticholinergic medications. Clinical observations in children suggest that the use of anticholinergic medications may contribute to delirium in children on prolonged mechanical ventilation^13^. Although anticholinergics may impair verbal learning and declarative memory, the potential adverse effects of anticholinergics are rarely considered in the clinical setting^14^. Studies evaluating the prevalence of anticholinergic burden in the pediatric population are also lacking^15^. Adults are at risk of experiencing multiple comorbidities and hence also at risk of polypharmacy and thus anticholinergic burden. A recent study of over fifty-thousand patients, who were aged 55 years and above, offered compelling evidence of a link between anticholinergic medication exposure and dementia risk in later life^8^. These findings highlight the need to include children and adults below 65 years in studies of anticholinergic burden since there is currently a poor understanding of the true anticholinergic burden, along with its clinical consequences, across the entire population.

The full extent of anticholinergic burden across the entire population is currently poorly understood. This study evaluates the anticholinergic burden in the Slovenian population by analyzing health claim data of medication prescriptions issued to outpatients in 2018, thus making an important contribution to bridging current gaps in understanding. Anticholinergic burden was evaluated using the anticholinergic cognitive burden (ACB) scale, which categorizes medications on a scale of 0 to 3, according to their anticholinergic properties. ACB score 0 means no anticholinergic effect, ACB score 1 represents possible anticholinergic effect, ACB scores 2 and 3 mean definite anticholinergic effect^16,17^. For each patient, anticholinergic burden (total ACB score) was also calculated.

## Results

### Study population characteristics

A total of 16.869.864 prescriptions were prescribed to 1.474.864 outpatients in Slovenia in 2018. The average patient age was 46.9 years (SD 24.1, median 50 years) and 56% of patients were female. An average of 11.4 (SD 13.1) prescriptions for 4.9 (SD 4.2) different medications were prescribed per patient in Slovenia in 2018.

### Most frequently prescribed anticholinergic medications

**Figure 1:**
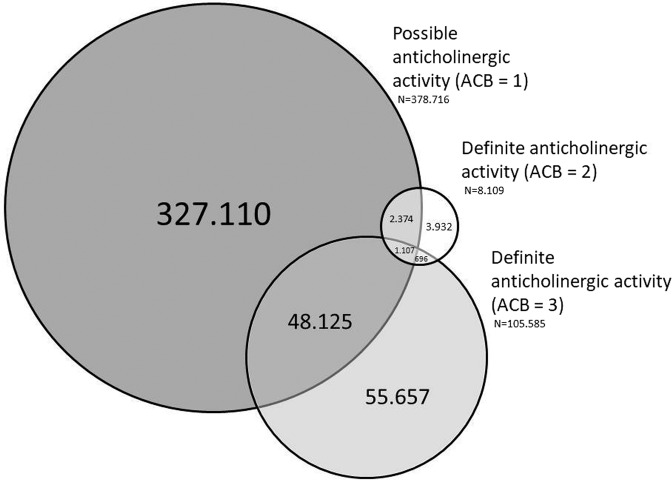
Most frequently prescribed medications with ACB score ≥1. The numbers represent patients with prescribed ACB medications.

Figure 1 presents the number of patients who received prescriptions for medications with anticholinergic activity. Approximately two thirds of patients, who were exposed to anticholinergic burden, received prescriptions with ACB score 1 and one quarter of patients received prescriptions with ACB score 3. A substantial number of patients received both prescriptions with ACB score 1 and ACB score 3. Prescriptions with ACB score 2 were seldom, also because few medicines are categorized as ACB score 2 in the ACB scale. Of note, patients presented as receiving medications with ACB score 1 only could have been prescribed multiple different medications with such score and hence still be exposed to a significant anticholinergic burden (total ACB score 3 or higher).

**Figure 2:**
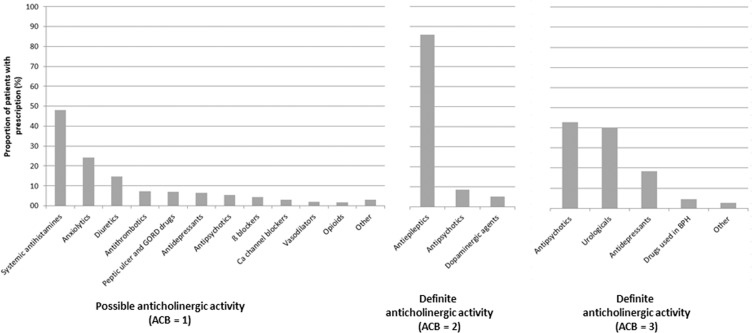
Most frequently prescribed pharmacological groups of medications with ACB score ≥1.

The most frequently prescribed medication groups with anticholinergic activity are presented in Fig. 2 and the medications are listed in Table 2. The two most frequently prescribed medication groups with ACB score 3were antipsychotics and medications for urinary diseases (42.8% and 40.2% of the study population, respectively). The three most frequently prescribed medications were quetiapine (antipsychotic), trospium (for urinary problems) and paroxetine (antidepressant), with trospium and quetiapine being prescribed to more than 60% of all older adults and more than 50% of adults who were prescribed any anticholinergic medications. For children, trospium and atropine (ophthalmic form) were the most frequently prescribed medications, comprising more than 80% of all children with prescriptions for medications with the highest anticholinergic activity.

The majority of patients with prescriptions for medications with ACB score 2 received antiepileptics (87.3% of all patients being prescribed medications with ACB score (2), namely carbamazepine (72.4%) and oxcarbazepine (14.9%). Only two other medications with ACB score 2 were prescribed in Slovenia in 2018: amantadine (8.8% of patients) and levopromazine (5.3% of patients).

Prescribed medications with ACB score of 1 belonged to a diverse range of ATC classifications. Overall, the most frequently prescribed were systemic first-generation antihistamines (48.0% of patients), followed by anxiolytics (24.0% of patients) and diuretics (14.6% of patients). Systemic antihistamines were the most commonly prescribed medication to children (95% of all children who were prescribed medications with ACB score 1). Adults and older adults were prescribed a wide range of medicine groups, including diuretics, antithrombotics (warfarin), β-blockers and medications for acid-related disorders.

### Anticholinergic burden

Approximately one third of study participants (439.001 patients, 29.8% of study population) were prescribed medications with anticholinergic activity and were thus exposed to anticholinergic burden (total ACB score ≥1). The average age of patients exposed to anticholinergic burden was 54 years and 59% of patients were female. A total of 7.6% of participants were exposed to clinically significant anticholinergic burden (total ACB score ≥3). These patients were on average prescribed 5.5 prescriptions for anticholinergic medications per year. Of the patients with any anticholinergic burden as well as in the group exposed to clinically significant anticholinergic burden, approximately half were adults (49.4% and 54.6%, respectively). The characteristics of the patients with any anticholinergic burden and of patients with clinically significant anticholinergic burden are presented in Table 1.

**Table 1:** Characteristics of total study population and of patients exposed to anticholinergic burden.

	ALL	Total ACB score ≥ 1	Total ACB score ≥ 3
Number of patients (%)	1.474.864 (100)	439.001 (29.8)	111.491 (7.6)
Female patients (%)	814.252 (56)	260.501 (59)	69.353 (62)
Age, years (SD)	46.9 (24.1)	53.8 (24.2)	59.1 (19.9)
Number of prescriptions per patient (SD)	11.4 (13.1)	19.6 (16.9)	25.3 (19.2)
Number of different medications prescribed per patient* (SD)	4.9 (4.2)	7.8 (5.1)	9.4 (5.6)
Number of prescriptions with ACB per patient (SD)	1.0 (2.5)	3.3 (3.6)	5.5 (5.3)
Number of different medications with ACB prescribed per patient* (SD)	0.4 (0.8)	1.4 (0.7)	1.9 (1.1)
**Number of patients per age groups (%)**			
children (≤18 years)	239.287 (16.2)	51.993 (11.8)	2.898 (2.6)
adults (19–64 years)	840.361 (57.0)	216.670 (49.4)	60.921 (54.6)
older adults (≥65 years)	395.216 (26.8)	170.338 (38.8)	47.672 (42.8)

*Different medications represent different ATC groups. ACB – anticholinergic cognitive burden. SD – standard deviation. Total ACB score ≥1 represents anticholinergic burden. Total ACB ≥ 3 represents clinically significant anticholinergic burden.

Examining the different age groups, anticholinergic medications were prescribed most frequently to older adults (43.1% of all older adults), followed by adults (25.8% of all adults) and least frequently to children (20.7% of all children). Although older adults were most likely to be exposed to clinically significant anticholinergic burden (total ACB score ≥3; 12.1% of older adults), the burden was also considerable among adults (7.3% of adults). Table 3 gives detailed information on the anticholinergic burden in the three age groups.

**Table 2:** List of most frequently prescribed medications with ACB score.

ACB score 1 (% of patients with prescription)				
	All (N = 378.716)	Older adults (N = 148.706)	Adults (N = 180.413)	Children (N = 49.597)
loratadine	**26.3**	**11.8**	**26.1**	**70.8**
alprazolam	**17.1**	**19.7**	**19.5**	0.3
furosemide	**14.6**	**32.0**	4.3	<1.0
desloratadine	**11.9**	4.7	**17.4**	**14.1**
warfarin	**7.2**	**16.2**	1.9	<1.0
diazepam	**7.0**	**7.2**	**8.4**	<1.0
ranitidine	**7.0**	**7.2**	**8.4**	<1.0
cetirizine	**6.6**	3.8	**7.7**	**11.3**
metoprolol	3.7	**6.9**	2.1	<1.0
risperidone	3.2	4.5	2.5	1.6
levocetirizine	3.0	<1.0	4.2	3.8
nifedipine	2.9	5.4	1.7	<1.0
trazodone	3.0	2.8	4.1	<1.0
venlafaxine	2.0	1.4	3.0	<1.0
isosorbide mononitrate	2.0	4.8	<1.0	<1.0
bupropion	<1.0	<1.0	2.2	<1.0
aripiprazole	<1.0	<1.0	1.7	<1.0
haloperidol	<1.0	1.9	<1.0	<1.0
fentanyl	<1.0	2.3	<1.0	<1.0
captopril	<1.0	2.0	<1.0	<1.0
**ACB score 2 (% of patients with prescription)**				
	**All (N = 8.109)**	**Older adults (N = 3.095)**	**Adults (N = 4.694)**	**Children (N = 320)**
carbamazepine	**72.4**	**74.9**	**73.3**	**36.3**
oxcarbazepine	**14.9**	**11.0**	**14.1**	**64.4**
levomepromazine	**8.8**	**7.5**	**10.4**	0
amantadine	**5.3**	**7.5**	4.2	0
**ACB score 3 (% of patients with prescription)**				
	**All (N = 105.585)**	**Older adults (N = 43.901)**	**Adults (N = 58.837)**	**Children (N = 2.847)**
quetiapine	**31.5**	**41.2**	**25.3**	**8.4**
trospium	**31.0**	**21.2**	**38.0**	**39.8**
paroxetine	**13.5**	**10.2**	**16.6**	1.3
olanzapine	**8.6**	**6.0**	**10.8**	3.7
solifenacin	**6.1**	**9.3**	3.9	0.3
amitriptyline	4.9	4.7	**5.2**	0.6
tamsulosin and solifenacin	4.6	**7.4**	2.7	0
atropine	2.7	1.8	1.5	**42.8**
clozapine	2.6	1.6	3.6	0.1
fesoterodine	1.6	1.3	0.6	0.2
darifenacin	1.5	2.6	0.8	0
tolterodine	0.7	0.9	0.3	4.1
scopolamine	<0.1	<0.1	<0.1	0

*Numbers in bold represent prevalence above 5%.

Of note: Each patient may have received prescriptions for two medications with mild anticholinergic burden in 2018, eg. loratadine and alprazolam, and therefore appeared in the calculation twice. Hence, the percentage sum in each category may be higher than 100.

**Table 3:** Anticholinergic burden in different age groups.

	Children	Adults	Older adults
Number of patients	239.287	840.361	395.216
Gender, % female	49	56	58
Age, years (SD)	7.9 (5.4)	44.7 (12.7)	75.4 (7.71)
Number of patients with total ACB score ≥1 within age group (%)	51.993 (20.7)	216.670 (25.8)	170.338 (43.2)
Number of patients with total ACB score ≥3 within age group (%)	2.898 (1.2)	60.921 (7.3)	47.672 (12.1)
Average total ACB score, mean (SD)*	1.13 (0.50)	1.79 (1.35)	1.85 (1.29)

*Data are presented for patients with CAB ≥ 1. ACB –anticholinergic cognitive burden.

## Discussion

One third of all study participants that received prescription medications in 2018 were prescribed at least one medication with anticholinergic burden. 7.6% of the study participants were exposed to clinically significant anticholinergic burden. Of these, half were adult patients. Half of the older adults and one quarter of adults and children were prescribed medications that have an anticholinergic burden. The medications with the highest anticholinergic burden included frequently prescribed medications, such as antipsychotics, antidepressants, and medications to treat urinary disease, while medications with moderate and mild anticholinergic burden belonged to a diverse range of therapeutic groups.

A limited number of previous studies report national prevalence of anticholinergic burden. Landi *et al*., who analyzed the anticholinergic burden of frail older adults living in nursing homes, reported 50% prevalence^12^. Two other studies, performed in Norway and in the USA, respectively, reported that up to 50% of older adults receive at least one medication with anticholinergic burden^3,10^; our study has a similar finding. A recent Slovenian study by Gorup *et al*., which investigated the anticholinergic burden in older primary care patients, reported that at least one anticholinergic medication was prescribed to 12.5% of patients; our study found considerably more patients receiving medications with anticholinergic burden. The difference in findings might be due to (i) the use of different scales to evaluate anticholinergic burden (Duran scale^18^ vs ACB scale), (ii) different participant population sizes (622 vs 439.001), or (iii) the inclusion of per needed medications in our study. On the other hand, in agreement with our findings, Gorup *et al*. reported that psychotropic medications were the most frequently prescribed medications with anticholinergic burden^19^. Other studies report higher anticholinergic burden prevalence; these studies are generally performed in clinical settings where the patient population is more vulnerable due to being older and having multiple comorbidities.

Studies of the anticholinergic burden in children and adults under 65 years are rare. Our study reveals that approximately one quarter of adults and children are prescribed anticholinergic medications, in agreement with the reported prevalence of anticholinergic medication prescriptions for children undergoing psychiatric treatment in the USA^14^. Worryingly, it has been shown that children may be more prone to adverse effects of anticholinergics^13^. Our study shows that up to 20% of children receiving prescription medications are exposed to anticholinergic burden. The majority of the prescribed anticholinergic medications are systemic antihistamines. These medications are used for numerous indications, such as allergic rhinitis and atopic eczema. For many, medications with local route of application may be used, which have little or no systemic effects^2^. Nonetheless, loratadine and desloratadine, the most frequently prescribed systemic antihistamine in our study, have only potential anticholinergic activity (ACB score 1). The use of medications with higher anticholinergic activity among children was significantly lower.

In our study, of all patients being exposed to anticholinergic burden, more than half were adults; this is an alarming result in the context that the anticholinergic burden is rarely studied in this age group. Also worrying is the prevalent use of antipsychotics by adults due to the association between anticholinergic burden and both adverse cognitive and physical function^20^. Other more recent findings associate long-term use of anticholinergic medications with increased dementia risk^4,8^. Taken together, these findings support the importance of appropriate primary prevention against cognitive decline and the avoidance of prescribing anticholinergic medication except in unavoidable circumstances.

Our results are most alarming in terms of potentially inappropriate prescribing. The majority of medications that fall into the group ACB of 3 are not recommended to be prescribed in the patient populations that they are usually prescribed for. For example, in our study, quetiapine (ACB of 3) was one of the most frequently prescribed medicines in both adult and older adults participants. In Slovenia, quetiapine is frequently prescribed in low doses for the *off-label* treatment of insomnia, despite a systematic review reporting that the benefits of using quetiapine to treat insomnia do not outweigh the risks^21^. Indeed, caution is needed when prescribing quetiapine for older adults since, for this age group, the average plasma clearance of quetiapine can be decreased up to 50%^2^. According to the Beers criteria, quetiapine should be prescribed at the lowest possible dose and for the shortest possible time^22^. Olanzapine, the fourth most frequently prescribed medicine with ACB of 3, also produces more pronounced anticholinergic side effects in older adults, such as hallucinations, incontinence and falls, and it should be prescribed with caution^2^. Similarly, the most commonly prescribed medications to children are, in the majority of cases, contraindicated. According to the SmPC’s of trospium, tolterodin and quetiapine, none of them are recommended to be prescribed for children due to lack of data confirming their safety; these medications are commonly prescribed for children *off-label*^2,23–25^. In the context that the prescription of these medications is sometimes unavoidable, caution and strict monitoring of adverse effects is necessary.

Our study has a number of important limitations that should be noted when interpreting the results. Anticholinergic burden was evaluated using the anticholinergic cognitive burden (ACB) scale. Many other scales are available, and they are not equivalent; thus, our results are not directly comparable with studies that do not also use the ACB scale. Nevertheless, since the ACB scale is the most frequently validated scale, it provides a robust evaluation for our study^26^. The ACB scale evaluates medicines that are likely to have a negative impact on cognition^3^. Other studies that use the ACB scale show that use of medications with definite anticholinergic burden (ACB of 3) is not only a predictor of cognitive impairment in older people, but also impairs physical function^7^. In light of recent findings regarding the relationship between exposure to anticholinergic medications and dementia, it is advantageous to use a scale that considers cognitive decline. Furthermore, ACB score 1 represent a possible anticholinergic effect, based on *in vitro* data and not supported by clinical evidence, hence the actual anticholinergic burden of these prescription medications may be questionable. Our use of a database that details medication prescriptions imposes certain limitations. The prevalence of anticholinergic burden was determined by the day the medication was dispensed rather than the day the patient took the medication, for which there was no data. Indeed, the patient may not have taken the medication at all since the database did not include information on patient adherence. Furthermore, many of the medications reported may be used per needed only; our results may, therefore, be exaggerated. In contrast, our study participants may have been using non-prescription (OTC) medications with anticholinergic burden; these medications would not have been included in the study database, resulting in an underestimation of the overall burden. Our results are, therefore, incomplete and should be further validated in the clinical setting, ideally in combination with clinical outcomes. Nevertheless, our large and diverse study population offers important methodological strengths, which are difficult to obtain in a clinical study design.

From a clinical perspective, our study highlights the need to increase the awareness of potential side effects when prescribing medications with anticholinergic burden. Since many medications prescribed for psychiatric indications have an anticholinergic burden, it is important that prescribing physicians are aware of potential side effects, such as cognitive impairment and delirium, and to seek to distinguish these side effects from true disease symptoms. Deprescribing medications that have anticholinergic burden in an effort to reduce side effects may be beneficial, especially in vulnerable populations, such as older adults and patients with polypharmacy, or decreased renal/hepatic function. Many of the often prescribed anticholinergic medications also have safer alternatives. Physicians however must carefully consider risks and benefits for individual patients before deprescribing in order to avoid withdrawal symptoms and diseases relapse^26,27^.

In summary, our study shows that, in Slovenia, one in three people receiving prescription medications were prescribed at least one medication with anticholinergic burden in 2018. Approximately 8% of the study population received a clinically significant anticholinergic burden, of whom half were adults. The medications with the highest anticholinergic burden (ACB of 3) belonged to diverse therapeutic groups including antipsychotics, antidepressant, and medications to treat urinary diseases. Our results call for greater awareness of potential adverse effects from medications that have anticholinergic burden in all age groups.

## Methods

### Study design

This retrospective cross-sectional study analyzed health claims data regarding all dispensed outpatient prescriptions, in 2018, specifically of medications that have a known anticholinergic burden.

### Data sources

Data detailing the health claims of medications that were prescribed and dispensed to outpatients in Slovenia in 2018 were obtained from a national database held by the Health Insurance Institute of Slovenia. The database used belongs to the National Insurance Institute of Slovenia and is not publicly available. The National Insurance Institute of Slovenia has provided permission to use the data from the database for the purpose of this study.

The Slovenian healthcare system provides statutory health insurance for the entire Slovenian population (approximately two million people), and the insurance fund is managed by the Health Insurance Institute of Slovenia. The health claims database details all publicly funded outpatient prescriptions dispensed in Slovenia, excluding over-the-counter medications, medications prescribed in hospitals, and private (out-of-pocket) prescriptions. Private (out-of pocket) prescriptions account for less than 5% of all outpatient prescriptions in Slovenia^28^. The Slovenian healthcare system and health claims data have been described in detail elsewhere^28–31^.

All data used in this study were anonymized; the allocation of unique patient identifiers allowed analysis of data from individual patients. Patient-specific variables were gender and year of birth. Prescription variables included dispensed medications, coded according to the Anatomical Therapeutic Chemical (ATC) classification, and prescription date. Since the database did not include information regarding therapeutic indication or medication dose, it was not possible to investigate any relationship between specific clinical information and anticholinergic burden.

### Anticholinergic burden

Anticholinergic burden was evaluated using the anticholinergic cognitive burden (ACB) scale^16,17^. This scale is widely recognized and validated for adverse anticholinergic outcomes and it was also applicable to our database, since the data on drug dosing was not available^26,32,33^. The ACB scale was first published in 2008 and updated in 2012^16,17^. Firstly, a list of possible anticholinergic medications was developed from a systematic review of published literature on anticholinergic effects of medications; the list of identified medications was further evaluated and categorized by a multi-disciplinary panel of expert clinicians. The final ACB scale categorizes medications on a scale of 0 to 3, according to their anticholinergic activity. Detailed scoring uses the following criteria: (i) ACB score of 1 (possible anticholinergic effect) requires “evidence from *in vitro* data that the medication has antagonist activity at muscarinic receptors”, (ii) ACB score of 2 (definite anticholinergic effect) requires “evidence from literature, prescriber’s information, or expert opinion of clinical anticholinergic effect”, (iii) ACB score of 3 (definite anticholinergic effect) requires “evidence from literature, prescriber’s information, or expert opinion of the medication causing delirium”. All other medications have a score of 0^16^. The total anticholinergic burden of a patient is calculated by summing the ACB scores from all the medications that patient receives concomitantly: anticholinergic burden ≥3 is considered clinically significant^17,34^. We coded each medication in the health claims database according to the ACB scale and then calculated the anticholinergic burden for each patient in the study (study participant), ready for further analysis. All methods were performed in accordance with relevant guidelines and regulations.

### Study participants

All outpatients who were dispensed at least one prescription medication in 2018 were included in the study. These outpatients were categorized into two groups: (i) outpatients with ACB = 0 (patients without anticholinergic medications) and (ii) outpatients with ACB ≥ 1 (patients with at least one anticholinergic medication). Another subgroup was created, namely outpatients with ACB ≥ 3, which is considered a clinically significant score^17,34^. These subgroups of patients were further analyzed according to gender and age. Ethical approval was not required, since the data for this study were anonymized.

### Statistical analysis

The unit of analysis was a participant. Anticholinergic burden was calculated for each study participant. The number of participants with no anticholinergic burden (total ACB score of 0), with anticholinergic burden (total ACB score ≥1) and with clinically significant anticholinergic burden (total ACB score ≥3) were presented according to patient gender and age, both in terms of absolute number and percentage of total study population. Participants were categorized into three age-based groups: (i) children (≤18 years), (ii) adults (19–64 years), and (iii) older adults (≥65 years). Average anticholinergic burden in each group was also calculated.

The most commonly prescribed medications with anticholinergic activity (ACB score ≥1) were identified and presented according to their ATC classification and age group. The prescription frequency for each medication or medication group (ATC level 3) was defined as the number of patients being prescribed that medication or medication group. All statistical analyses were performed using IBM SPSS Statistics 25.0.
